# Intentional asymmetric balancing in robotic‐assisted valgus TKA: Early outcomes and intraoperative laxity changes

**DOI:** 10.1002/jeo2.70810

**Published:** 2026-06-22

**Authors:** Stefano G. Petrillo, Damiano Ardiri, Matteo Marullo, Giampaolo Giordano, Sergio Romagnoli

**Affiliations:** ^1^ Prosthetic Surgery Centre, IRCCS Galeazzi Sant'Ambrogio Hospital Milan Italy

**Keywords:** laxity, robotic‐assisted TKA, semi‐personalized alignment, soft‐tissue balancing, valgus knee

## Abstract

**Purpose:**

To evaluate early patient‐reported outcome measures (PROMs), radiographic alignment changes, intraoperative laxity changes and complications in a retrospective series of constitutional valgus knees treated with robotic‐assisted total knee arthroplasty (TKA) using an intentional asymmetric balancing strategy and a semi‐personalized (SP) alignment technique.

**Methods:**

This retrospective single‐centre cohort included consecutive patients with end‐stage valgus osteoarthritis (OA) and constitutional valgus morphotypes (coronal plane alignment of the knee III/VI) treated with imageless robotic‐assisted TKA and a minimum follow‐up of 12 months. The balancing workflow intentionally accepted mild residual lateral laxity. Medial and lateral compartment laxity (mm) were quantified intraoperatively at 0° and 90°. PROMs (Knee Society Score [KSS] and Forgotten Joint Score [FJS]), range of motion (ROM) and hip–knee–ankle angle (HKA) were assessed preoperatively and at the latest follow‐up. Complications were recorded.

**Results:**

Fifty‐eight patients were included at a mean follow‐up of 26.5 ± 15.0 months. KSS Knee improved from 58.3 ± 9.4 to 87.9 ± 11.0 (mean change +29.6 points, 95% confidence interval [CI] = 26.0–33.2; *p* = 0.00002) and KSS Function from 58.8 ± 15.0 to 82.4 ± 15.7 (mean change +23.6 points, 95% CI = 18.8–28.4; *p* = 0.00004). Flexion contracture improved from 3.9 ± 4.0° to 0.6 ± 1.2° (mean change −3.3°, 95% CI = −4.3 to −2.3; *p* = 0.00010), while flexion increased from 101.4 ± 6.0° to 126.8 ± 8.6° (mean change +25.4°, 95% CI = 23.2–27.6; *p* = 0.00001). HKA improved from 6.2 ± 4.5° valgus to 2.1 ± 1.8° valgus (mean change −4.1°, 95% CI = −5.2 to −3.0; p = 0.00030). Lateral laxity decreased in extension from 4.5 ± 1.7 to 2.5 ± 1.3 mm (mean change −2.0 mm, 95% CI = −2.7 to −1.3; *p* = 0.001) and at 90° from 5.3 ± 2.0 to 1.9 ± 1.2 mm (mean change −3.4 mm, 95% CI = −4.3 to −2.5; *p* = 0.0008), with smaller medial reductions. Post‐operative complications occurred in four patients (6.9%) (deep vein thrombosis, transient peroneal neuropraxia, superficial wound issue and one manipulation under anaesthesia); there were no pulmonary emboli, periprosthetic joint infection or revision procedures.

**Conclusion:**

In this retrospective single‐surgeon series of constitutional valgus knees treated with robotic‐assisted TKA, intentional asymmetric balancing was associated with good early clinical outcomes, correction towards less valgus alignment, and a reproducible reduction in lateral laxity, with a low complication rate. These findings support the feasibility of this measurable laxity‐guided strategy, but do not establish superiority over alternative balancing philosophies.

**Level of Evidence:**

Level III, retrospective cohort.

**Abbreviations**
CPAK: coronal plane alignment of the knee
FJS: Forgotten Joint Score
HKA: hip–knee–ankle angle
KSS: Knee Society Score
OA: osteoarthritis
PROMs: patient‐reported outcome measures
ROSA: robotic surgical assistant
SP: semi‐personalized
TKA: total knee arthroplasty

## INTRODUCTION

Contemporary total knee arthroplasty (TKA) is still challenged by the tension between neutral alignment targets and the goal of reproducing physiologic knee function. Native tibiofemoral motion is inherently asymmetric, with compartment‐specific behaviour and a centre of rotation located predominantly on the lateral side during gait rather than perfectly symmetric mediolateral motion, suggesting that ‘perfectly symmetric’ mechanics may not represent normal knee physiology [8, 11, 19]. In addition, static coronal alignment after TKA does not reliably predict dynamic loading or gait alignment, highlighting the limits of purely radiographic targets [10, 22, 24]. From a clinical standpoint, residual instability, particularly in mid‐flexion, remains a relevant cause of symptoms and failure [31]. The traditional paradigm of symmetric rectangular gap balancing has therefore been questioned [23], and emerging concepts emphasize compartment‐specific laxity targets and the potential benefit of preserving controlled asymmetry, especially in flexion rather than forcing symmetry at all costs [9, 21]. While kinematic alignment may improve kinematics compared with strict mechanical alignment in selected scenarios [14, 35], valgus phenotypes raise specific concerns and may require restricted, phenotype‐aware strategies [27, 28, 33, 36, 37, 38]. Robotic‐assisted TKA provides a unique opportunity to operationalize these principles by improving component placement accuracy and enabling objective, repeatable quantification of gaps and laxity throughout the range of motion [1, 2, 5, 6, 15, 17, 25, 26, 32]. This is particularly relevant in valgus knees, in which lateral soft‐tissue imbalance is common, although some cases may also present with lateral tightness depending on deformity pattern, wear, and soft‐tissue contracture. In constitutional valgus phenotypes, however, excessive lateral laxity often represents a major balancing problem and was the specific focus of the present workflow. Therefore, the purpose of this retrospective cohort study was to evaluate early patient‐reported outcome measures (PROMs), radiographic alignment changes, and intraoperative compartmental laxity changes following an intentional asymmetric soft‐tissue balancing strategy in robotic valgus TKA. We hypothesized that this approach would be associated with good early clinical outcomes and radiographic alignment changes towards less valgus hip–knee–ankle angle (HKA), with a measurable reduction of excessive lateral laxity while preserving mild, intended mediolateral asymmetry [15, 17, 21].

## OBJECTIVES AND HYPOTHESIS

The primary aim of this study was to assess early PROMs and intraoperative compartmental laxity changes following an intentional asymmetric soft‐tissue balancing strategy in robotic‐assisted valgus TKA. The secondary aim was to evaluate radiographic alignment changes (HKA, medial proximal tibial angle [MPTA] and lateral distal femoral angle [LDFA]) from preoperative to post‐operative radiographs. We hypothesized that intentional asymmetric balancing would produce favourable early PROMs and reduce excessive lateral laxity while preserving mild, intended mediolateral asymmetry [15, 17, 21].

## MATERIALS AND METHODS

### Reporting guideline

This study was conducted and reported in accordance with the STROBE statement for observational studies.

### Study design, setting and dates

This was a single‐centre retrospective cohort study (Level of evidence III). Consecutive patients undergoing robotic‐assisted total knee arthroplasty (RA‐TKA) at the Joint Replacement Department, IRCCS Galeazzi–Sant'Ambrogio Hospital (Milan, Italy) were screened for eligibility. The study period ranged from 1 July 2022 to 1 February 2025. Post‐operative full‐length weight‐bearing radiographs were routinely obtained at 3 months, while clinical assessment and PROMs were collected preoperatively and at a minimum of 12 months after surgery.

### Participants

Inclusion criteria were: (1) end‐stage valgus knee osteoarthritis (OA; Kellgren–Lawrence Grade III and IV), (2) constitutional valgus morphotypes coronal plane alignment of the knee (CPAK) III or VI on preoperative full‐length weight‐bearing radiographs, and (3) minimum follow‐up ≥12 months. Exclusion criteria were: varus knee OA, active or prior infection, previous ipsilateral knee fractures (including valgus deformity secondary to tibial plateau fracture sequelae), revision procedures, missing post‐operative radiographs, incomplete robotic surgical reports, use of a different implant design, use of a different alignment technique and inability to complete clinical questionnaires.

## SURGICAL TECHNIQUE AND INTENTIONAL ASYMMETRIC BALANCING PROTOCOL

Following anatomical registration and verification, intraoperative laxity acquisition was performed according to the standard imageless robotic surgical assistant (ROSA) workflow, with the patella reduced in all measurements. An initial acquisition was obtained after exposure, osteophyte removal, tracker fixation, and completion of registration, before bone resections and balancing adjustments, in order to characterize the native soft‐tissue envelope. Manual varus and valgus stress were applied by the operating surgeon (S.G.P.) following the standard ROSA acquisition sequence, and medial and lateral compartment gaps were recorded directly from the robotic interface in millimetres at 0° of extension and 90° of flexion. A final acquisition was then obtained, again with the patella reduced, after completion of the bone resections, implant planning adjustments, and insertion of the trial components, immediately before final implantation. The same acquisition sequence and stress‐testing manoeuvre were used in all cases to maximize measurement consistency.

## BONY ALIGNMENT PLAN: SEMI‐PERSONALIZED (SP) ALIGNMENT TECHNIQUE

The bony alignment plan followed an SP alignment technique. In this study, SP alignment referred to a hybrid strategy combining a standardized tibial cut with individualized femoral coronal planning and balancing‐driven adjustments according to each knee's soft‐tissue envelope. The tibial resection was planned perpendicular (90°) to the tibial mechanical axis in the coronal plane, with a posterior slope of 7° in the sagittal plane (modifiable up to 9° in selected cases), and a planned resection depth of 6–9 mm. The femoral plan targeted 1–3° of valgus relative to the femoral mechanical axis with 3° of flexion and a 9‐mm medial distal resection.

### Balancing strategy: Intentional mediolateral asymmetry

Rather than enforcing perfectly symmetric rectangular gaps, the workflow intentionally aimed for a controlled mediolateral asymmetry, allowing the lateral compartment to remain slightly more lax than the medial compartment while avoiding excessive laxity. Extension balance was simulated on the planning interface with a target mediolateral gap difference of <3 mm. Flexion balance was then optimized by adjusting femoral component external rotation to reproduce the extension gaps, maintaining a tolerance of ≤3 mm per compartment. For case‐level target‐achievement analysis, a knee was considered within target in extension when the final lateral‐minus‐medial gap difference at 0° was ≥0 and <3 mm, within target at 90° when the final lateral‐minus‐medial gap difference at 90° was ≥0 and <3 mm, and within target at both positions when both criteria were simultaneously satisfied.

In cases with severe preoperative flexion contracture, distal femoral resection was increased by 1–2 mm (maximum total distal resection 11 mm). In the presence of a tight flexion gap, posterior tibial slope was increased (up to 9°) and/or the femoral component was downsized by one size, at the surgeon's discretion. Patellar resurfacing was reserved for cases with severe trochlear or patellar osteochondral loss.

### Outcomes and measurements

#### Clinical outcomes

PROMs included the Knee Society Score (KSS), collected preoperatively and at ≥12 months follow‐up. The Forgotten Joint Score (FJS) was collected at follow‐up only. Range of motion (ROM) was assessed clinically.

### Radiographic outcomes

Full‐length weight‐bearing AP radiographs were obtained preoperatively and at 3 months post‐operatively to measure HKA and to classify CPAK morphotype. Post‐operative component coronal angles (MPTA and LDFA) were measured on full‐length weight‐bearing radiographs and compared with preoperative values to describe radiographic alignment changes. Radiographic measurements were independently performed by three authors (S.G.P., D.A. and G.G.) using Sectra IDS7 (Sectra). The observers were blinded to each other's measurements and to clinical outcomes at the time of radiographic assessment. Interobserver reliability analysis was performed.

### Intraoperative laxity outcomes

Medial and lateral compartment gaps at 0° of extension and 90° of flexion were analyzed by comparing the initial acquisition before bone resections and balancing adjustments with the final acquisition after completion of the balancing workflow, as described above.

#### Adverse events

Intraoperative complications, post‐operative complications (including thromboembolic events, infection, stiffness and periprosthetic fracture), reoperations and revision procedures were recorded from clinical charts and institutional records.

#### Bias

To reduce selection bias, all eligible cases within the study period were included as a consecutive series. Surgical technique, implant design and robotic platform were standardized (single surgeon, single implant and single robotic system). When applicable, clinical and radiographic assessments were performed by an examiner not involved in the surgical procedure.

#### Study size

No a priori power analysis was performed due to the retrospective design. The sample size reflected all consecutive patients meeting eligibility criteria during the study period.

#### Ethics approval

This retrospective study was approved by the Comitato Etico Territoriale Lombardia 1 (CET Lombardia 1) (meeting date: 4 February 2026), promoted by IRCCS Ospedale Galeazzi–Sant'Ambrogio, protocol code: ALLCCP. The study was conducted in accordance with the Declaration of Helsinki and relevant local regulations. Due to the retrospective design and use of de‐identified data, the requirement for written informed consent was waived by the Ethics Committee. Statistical analysis.

Statistical analysis was performed by a biomedical statistician using SAS software (Version 9.4; SAS Institute Inc.). Categorical variables were summarized as absolute and relative frequencies. Continuous variables were reported as mean ± standard deviation. Preoperative versus post‐operative PROMs, ROM, radiographic parameters and intraoperative laxity measures were compared using paired analyses. For normally distributed variables, paired *t* tests were used, and results were reported as mean changes with 95% confidence intervals (CIs) and exact *p* values. For variables with non‐normal distribution, Wilcoxon signed‐rank tests were used, and exact *p* values were reported. Subgroup comparisons between CPAK III and CPAK VI were considered exploratory because of the limited size of the CPAK VI subgroup and were analyzed using appropriate parametric or non‐parametric tests according to data distribution. Interobserver reliability for radiographic measurements was assessed using the intraclass correlation coefficient (ICC). Missing data were handled by complete‐case analysis; cases with missing post‐operative radiographs or incomplete robotic reports were excluded a priori per eligibility criteria. Statistical significance was set at *p* < 0.05.

Baseline demographic and radiographic characteristics are summarized in Table 1.

**Table 1:** Baseline demographic and radiographic characteristics (*n* = 58).

Variable	Value
Age (years), mean ± SD (range)	67.7 ± 7.4 (50–82)
Sex, *n* (%)	Male 12 (20%)/female 46 (80%)
BMI (kg/m^2^), mean ± SD (range)	28.6 ± 4.7 (19–39)
Kellgren–Lawrence grade, *n* (%)	III 14 (24%)/IV 44 (76%)
Preoperative HKA (° valgus), mean ± SD (range)	6.2 ± 4.5 (2–16)
Preoperative CPAK morphotype, *n* (%)	III 49 (84%)/VI 9 (16%)
Follow‐up (months), mean ± SD	26.5 ± 15.0

*Note*: Baseline demographics, osteoarthritis severity, preoperative coronal alignment and morphotype distribution of the included cohort.

Abbreviations: BMI, body mass index; CPAK, coronal plane alignment of the knee; HKA, hip–knee–ankle angle; SD, standard deviation.

## RESULTS

### Participant flow

A total of 202 patients undergoing robotic‐assisted TKA during the study period were screened for eligibility. Overall, 144 patients (71%) were excluded. Among excluded patients (*n* = 144), reasons for exclusion were: varus morphotype (*n* = 124; 86%), incomplete intraoperative robotic reports (*n* = 4; 3%), use of a different prosthetic design (*n* = 10; 7%), valgus deformity secondary to tibial plateau fracture sequelae (*n* = 3; 2%), and use of a mechanical alignment technique (*n* = 3; 2%). Consequently, 58 patients were included in the final analysis.

### Clinical and functional outcomes

At a mean follow‐up of 26.5 ± 15.0 months, significant improvements were observed in clinical scores and range of motion (Table 2). KSS Knee improved from 58.3 ± 9.4 to 87.9 ± 11.0 (mean change +29.6 points, 95% CI = 26.0–33.2; *p* = 0.00002), and KSS Function from 58.8 ± 15.0 to 82.4 ± 15.7 (mean change +23.6 points, 95% CI = 18.8–28.4; *p* = 0.00004). Flexion contracture improved from 3.9 ± 4.0° to 0.6 ± 1.2° (mean change −3.3°, 95% CI = −4.3 to −2.3; *p* = 0.00010), while flexion increased from 101.4 ± 6.0° to 126.8 ± 8.6° (mean change +25.4°, 95% CI = 23.2–27.6; p = 0.00001). Mean FJS at latest follow‐up was 94.1 ± 4.4.

**Table 2:** Preoperative versus post‐operative clinical outcomes (*n* = 58).

Outcome	Preoperative (mean ± SD)	Post‐operative (mean ± SD)	Mean change (95% CI)	Exact *p*
KSS Knee	58.3 ± 9.4	87.9 ± 11.0	+29.6 (26.0–33.2)	0.00002
KSS Function	58.8 ± 15.0	82.4 ± 15.7	+23.6 (18.8–28.4)	0.00004
FJS	n.a.	94.1 ± 4.4	n.a.	n.a.
Flexion contracture (°)	3.9 ± 4.0	0.6 ± 1.2	−3.3 (−4.3 to −2.3)	0.00010
Flexion (°)	101.4 ± 6.0	126.8 ± 8.6	+25.4 (23.2–27.6)	0.00001

*Note*: Changes in PROMs and range of motion from preoperative baseline to latest follow‐up. FJS was collected post‐operatively only.

Abbreviations: CI, confidence interval; FJS, Forgotten Joint Score; KSS, Knee Society Score; PROM, patient‐reported outcome measure; SD, standard deviation.

### Radiographic outcomes

Mean coronal alignment improved from a preoperative HKA of 6.2 ± 4.5° valgus to a post‐operative HKA of 2.1 ± 1.8° valgus (mean change −4.1°, 95% CI = −5.2 to −3.0; *p* = 0.00030). Interobserver reliability for radiographic measurements was excellent overall (ICC = 0.91, 95% CI = 0.84–0.95), with ICC values of 0.94 (95% CI = 0.89–0.97) for HKA, 0.90 (95% CI = 0.83–0.95) for MPTA and 0.88 (95% CI = 0.80–0.94) for LDFA. Morphotype‐specific radiographic outcomes are reported in Table 3.

**Table 3:** Radiographic outcomes (degrees).

Group	Parameter	Preoperative (mean ± SD)	Post‐operative (mean ± SD)	Mean change	95% CI	Exact *p*
Overall	HKA (° valgus)	6.2 ± 4.5	2.1 ± 1.8	−4.1	−5.2 to −3.0	0.00030
CPAK III	MPTA	89.2 ± 1.5	90.8 ± 1.4	+1.6	1.1–2.1	0.00003
CPAK III	LDFA	84.5 ± 1.5	89.1 ± 1.7	+4.6	4.0–5.2	0.00001
CPAK VI	MPTA	87.5 ± 1.3	90.2 ± 1.8	+2.7	1.0–4.4	0.00054
CPAK VI	LDFA	91.6 ± 1.3	92.4 ± 1.6	+0.8	−0.2 to 1.8	0.108

*Note*: Overall HKA change is reported with mean change and 95% confidence interval. Morphotype‐specific changes in MPTA and LDFA are reported as preoperative to post‐operative paired comparisons.

Abbreviations: CI, confidence interval; CPAK, coronal plane alignment of the knee; HKA, hip–knee–ankle angle; LDFA, lateral distal femoral angle; MPTA, medial proximal tibial angle; SD, standard deviation.

### Intraoperative compartmental laxity outcomes

Compared with the initial intraoperative acquisition, the final acquisition after completion of the balancing workflow showed a significant reduction in lateral compartment laxity while preserving mild residual mediolateral asymmetry (Table 4). In extension, medial gap decreased from 1.8 ± 0.8 to 1.3 ± 0.7 mm (mean change −0.5 mm, 95% CI = −0.8 to −0.2; *p* = 0.002) and lateral gap from 4.5 ± 1.7 to 2.5 ± 1.3 mm (mean change −2.0 mm, 95% CI = −2.7 to −1.3; *p* = 0.001). At 90° of flexion, medial gap decreased from 1.5 ± 0.7 to 0.7 ± 0.6 mm (mean change −0.8 mm, 95% CI = −1.2 to −0.3; *p* = 0.003) and lateral gap from 5.3 ± 2.0 to 1.9 ± 1.2 mm (mean change −3.4 mm, 95% CI = −4.3 to −2.5; *p* = 0.0008). Using the prespecified intraoperative target of a mild residual lateral‐greater‐than‐medial asymmetry with a final mediolateral gap difference <3 mm, 50 out of 58 knees (86.2%) were within target in extension and 48 out of 58 knees (82.8%) were within target at 90° of flexion. Overall, 45 out of 58 knees (77.6%) achieved the target at both positions (Table 5). Most out‐of‐target cases were due to persistent residual lateral excess rather than overcorrection.

**Table 4:** Intraoperative compartmental laxity before balancing and after final balancing (robotic measurements, mm).

Laxity	Initial acquisition (mean ± SD)	Final acquisition (mean ± SD)	Mean change (95% CI)	Exact *p*
Extension—medial	1.8 ± 0.8	1.3 ± 0.7	−0.5 (−0.8 to −0.2)	0.002
Extension—lateral	4.5 ± 1.7	2.5 ± 1.3	−2.0 (−2.7 to −1.3)	0.001
Flexion (90°)—medial	1.5 ± 0.7	0.7 ± 0.6	−0.8 (−1.2 to −0.3)	0.003
Flexion (90°)—lateral	5.3 ± 2.0	1.9 ± 1.2	−3.4 (−4.3 to −2.5)	0.0008

*Note*: Intraoperative medial and lateral compartment gaps quantified by the ROSA robotic system at 0° of extension and 90° of flexion at two predefined time points: initial acquisition before bone resections and balancing adjustments, and final acquisition after completion of the balancing workflow with trial components in place. All measurements were obtained with the patella reduced.

Abbreviations: CI, confidence interval; ROSA, robotic surgical assistant; SD, standard deviation.

**Table 5:** Target achievement at final acquisition (*n* = 58).

Outcome	*n*/*N*	%
Within target in extension (0°)	50/58	86.2
Within target at 90° of flexion	48/58	82.8
Within target at both positions	45/58	77.6

*Note*: A knee was considered within target in extension when the final lateral‐minus‐medial gap difference at 0° was ≥0 and <3 mm, within target at 90° when the final lateral‐minus‐medial gap difference at 90° was ≥0 and <3 mm, and within target at both positions when both criteria were simultaneously satisfied.

### Complications

Four patients (6.9%) experienced post‐operative complications during follow‐up, with no intraoperative complications, no periprosthetic joint infection, and no revision procedures at the latest follow‐up (Table 6). Observed events included one symptomatic deep vein thrombosis treated with therapeutic anticoagulation, one transient common peroneal nerve neuropraxia with complete recovery, one superficial wound complication treated nonoperatively and one case of post‐operative stiffness managed with manipulation under anaesthesia.

**Table 6:** Post‐operative complications, reoperations and revisions (*n* = 58).

Event	*n* (%)	Timing	Management	Outcome
Patients with ≥1 complication	4 (6.9%)	–	–	–
Intraoperative complications	0 (0%)	–	–	–
Symptomatic VTE (DVT; no PE)	1 (1.7%)	Post‐op Day 9	Therapeutic DOAC for 3 months; no IVC filter	Resolved; no recurrence
Transient common peroneal nerve palsy (neuropraxia)	1 (1.7%)	Immediate post‐op	AFO + physiotherapy; EMG at 6 weeks	Complete recovery by 3 months
Superficial wound complication (erythema/dehiscence)	1 (1.7%)	Post‐op Day 14	Local wound care + oral antibiotics	Healed; no deep infection
Stiffness requiring manipulation under anaesthesia (MUA)	1 (1.7%)	Post‐op Week 7	MUA	Flexion improved and maintained at follow‐up
Periprosthetic fracture	0 (0%)	–	–	–
Periprosthetic joint infection (PJI)	0 (0%)	–	–	–
Reoperation (any)	1 (1.7%)	Post‐op Week 7	MUA	No further procedures
Revision TKA (component exchange)	0 (0%)	–	–	–

*Note*: Post‐operative adverse events, reoperations, and revisions recorded during follow‐up.

Abbreviations: AFO, ankle‐foot orthosis; DOAC, direct oral anticoagulant; DVT, deep vein thrombosis; EMG, electromyography; PE, pulmonary embolism; TKA, total knee arthroplasty; VTE, venous thromboembolism.

### Exploratory subgroup analysis

Exploratory subgroup analyses showed no statistically significant differences between CPAK III and CPAK VI in post‐operative KSS Knee (*p* = 0.412), KSS Function (*p* = 0.563) or FJS (*p* = 0.618). Given the smaller size of the CPAK VI subgroup, reflecting the relative frequency of constitutional valgus morphotypes in routine practice, these comparisons should be interpreted as exploratory.

## DISCUSSION

### Key results

The main finding of this study is that intentional asymmetric soft‐tissue balancing in robotic‐assisted valgus TKA was feasible within a standardized robotic workflow and was associated with good early clinical outcomes, correction towards less valgus alignment, and a consistent reduction in lateral laxity. At a mean follow‐up of 26.5 ± 15.0 months, KSS Knee improved from 58.3 ± 9.4 to 87.9 ± 11.0 (mean change +29.6 points, 95% CI = 26.0–33.2; *p* = 0.00002) and KSS Function from 58.8 ± 15.0 to 82.4 ± 15.7 (mean change +23.6 points, 95% CI = 18.8–28.4; *p* = 0.00004). Flexion contracture improved from 3.9 ± 4.0° to 0.6 ± 1.2° (mean change −3.3°, 95% CI = −4.3 to −2.3; *p* = 0.00010), while flexion increased from 101.4 ± 6.0° to 126.8 ± 8.6° (mean change +25.4°, 95% CI = 23.2–27.6; *p* = 0.00001). Coronal alignment improved from 6.2 ± 4.5° valgus to 2.1 ± 1.8° valgus (mean change −4.1°, 95% CI = −5.2 to −3.0; *p* = 0.00030). Intraoperatively, the largest laxity reductions occurred laterally, with the lateral gap decreasing from 4.5 to 2.5 mm in extension (mean change −2.0 mm, 95% CI = −2.7 to −1.3; *p* = 0.001) and from 5.3 to 1.9 mm at 90° of flexion (mean change −3.4 mm, 95% CI = − 4.3 to −2.5; *p* = 0.0008), while medial changes were smaller. Final mediolateral laxity differences were approximately 1.2 mm in both extension and flexion, consistent with achievement of a controlled and reproducible asymmetric laxity pattern rather than full mediolateral symmetry. The overall post‐operative complication rate was 6.9%, with no intraoperative complications, no periprosthetic joint infection and no revision procedures at the latest follow‐up.

### Numeric comparison with the literature

Our findings are consistent with the concept that balanced knee arthroplasty does not necessarily require perfectly symmetric mediolateral laxity. Native knee motion is compartment‐specific [8, 11, 19], and the dogma of perfectly rectangular gaps has been increasingly questioned [23]. In this framework, our cohort showed a selective reduction of the excessive lateral laxity typical of valgus knees: lateral laxity decreased from 4.5 to 2.5 mm in extension (mean change −2.0 mm) and from 5.3 to 1.9 mm at 90° of flexion (mean change −3.4 mm), whereas medial changes were smaller (1.8–1.3 mm and 1.5–0.7 mm). These magnitudes are consistent with a purposeful asymmetric balancing strategy rather than an attempt to force symmetry.

Importantly, the final mediolateral laxity difference in our series was approximately 1.2 mm in both extension and flexion, placing both conditions within the <2.5 mm category proposed by the REAL (Robotic Evaluation of Articular Laxity) classification [17]. In the REAL study, mediolateral laxity difference was categorized as <2.5, 2.5–5 and >5 mm, and 93% of knees ended in the balanced area (Classes 1A/1B) at the end of the procedure [17]. In practical terms, our final values fall within this balanced band while still preserving a mild intended lateral‐greater‐than‐medial laxity.

When comparing early outcomes with robotic valgus cohorts using functional alignment, Gregori et al. reported in 58 valgus RA‐TKAs that 86.2% achieved a post‐operative coronal alignment within the safe zone (HKA = 177–183°), with radiographic HKA improving from 187° (approximately 7° valgus) to 181.1° (approximately 1.1° valgus) [12]. In the same cohort, KSS improved from 69.5 to 95 for the Knee component and from 65 to 94 for the Function component, and the median FJS at final follow‐up was 93 [12]. In our series, HKA improved from 6.2° valgus to 2.1° valgus and FJS at follow‐up was 94.1 ± 4.4; thus, the magnitude of valgus correction and the FJS values are comparable, whereas the absolute KSS values were somewhat lower, likely reflecting differences in baseline scores, implant design, balancing philosophy and follow‐up structure between cohorts.

For alignment boundaries under robotics, Karasavvidis et al. found in 234 cases that unrestricted kinematic alignment and strict mechanical alignment resulted in similar post‐operative arithmetic HKA, with most cases remaining within accepted coronal limits [16]. Although that cohort was not valgus‐specific, it supports the notion that robotic execution tends to keep final coronal alignment within accepted boundaries even when personalized alignment labels differ.

Finally, regarding radiographic accuracy with the ROSA platform, Rossi et al. reported that planned‐to‐measured differences for cuts and angles were generally below 1 mm or 1°, and that the average difference between planned HKA and measured HKA was 1.2 ± 1.1° without statistically significant deviation [25]. While our study focused on clinical and laxity endpoints rather than planned‐versus‐achieved component positioning, the final radiographic alignment in our series is consistent with the degree of precision reported for contemporary robotic workflows.

### Clinical implications

From a practical standpoint, the present data suggest that defining and reproducing compartment‐specific laxity targets in valgus TKA is feasible within a robotic workflow. In this workflow, the surgical aim was to reduce marked lateral laxity while preserving a small residual lateral‐greater‐than‐medial asymmetry, rather than forcing a perfectly rectangular gap. This concept is compatible with native compartment behaviour and with the growing view that balanced TKA does not necessarily imply strict mediolateral symmetry [11, 19, 21, 23]. Clinically, this is relevant because instability, particularly in mid‐flexion, is a recognized driver of symptoms, dissatisfaction, and failure after TKA [31].

In the present series, robotic assistance allowed this approach to be implemented using measurable intraoperative laxity data and a standardized acquisition workflow [15, 17, 20]. The marked reduction in lateral laxity reduced the mediolateral difference to approximately 1.2 mm in both extension and flexion, which aligns with the balanced area threshold proposed by robotic laxity classifications (< 2.5 mm) [17]. This provides a target range that is objective, communicable, and potentially auditable across surgeons and centres.

In valgus knees, where balancing can be challenging, an objective laxity‐guided strategy may help standardize intraoperative decision‐making and reduce reliance on purely subjective assessment. Whether it reduces the need for soft‐tissue release or improves stability compared with other strategies remains to be determined. This also frames alignment philosophy as a tool rather than an ideology: constrained personalized alignment concepts such as restricted KA, inverse KA, and functional alignment all share the goal of remaining within safe boundaries while optimizing function [12, 14, 18, 33, 36, 37, 38]. Given that static alignment alone does not reliably predict dynamic loading after TKA [22, 24], focusing on compartmental laxity may represent a more direct lever on perceived stability and functional confidence after surgery.

Finally, the combination of favourable early PROMs and consistent reduction in lateral laxity suggests that intentional asymmetric balancing is a reasonable strategy to investigate further in constitutional valgus phenotypes, where the surgical problem is often driven by lateral laxity and soft‐tissue behaviour rather than coronal axis correction alone. Future work should test whether this measurable laxity‐target strategy reduces mid‐flexion instability events and improves longer‐term survivorship compared with symmetric balancing or alternative alignment protocols [1, 2, 5, 6, 26, 31].

### Interpretation and future directions

Taken together, our results support the feasibility of a pragmatic strategy in valgus TKA in which reduction of excessive lateral laxity is prioritized over enforcement of perfectly symmetric gaps [9, 21, 23, 31]. This interpretation is aligned with the biomechanical premise that native knee motion is compartment‐specific [8, 11, 19] and with evidence that static alignment alone does not reliably predict dynamic loading and gait behaviour after TKA [3, 22, 24, 29, 30]. Robotic assistance may add value by enabling quantifiable and repeatable laxity targets during implantation, which can be documented and audited [15, 17], potentially addressing mechanisms linked to instability, particularly in mid‐flexion [31].

At the same time, the present study does not establish that this strategy is superior to symmetric balancing or to other phenotype‐aware alignment philosophies. Rather, it shows that the approach can be executed in a consistent manner, that its intraoperative laxity effects can be measured objectively, and that it was associated in this series with favourable early clinical outcomes and low short‐term complication rates. Future prospective studies should compare intentional asymmetric balancing with symmetric balancing or alternative alignment strategies in valgus phenotypes using standardized laxity acquisition and reporting frameworks, and should evaluate whether achieving objective laxity targets translates into lower rates of instability‐related symptoms, reoperations, and revision at longer follow‐up [1, 2, 5, 6, 15, 17, 26, 31].

### Generalisability

These findings are most applicable to constitutional valgus knees treated with an imageless robotic workflow and an ultracongruent cruciate‐retaining implant using a standardized medial approach. Extrapolation to other valgus subtypes, different levels of implant constraint, alternative surgical approaches, including lateral approaches, and other robotic platforms should therefore be made with caution and warrants dedicated validation [1, 2, 4, 5, 6, 7, 13, 26, 34].

### Limitations

A few limitations should be acknowledged. First, this was a retrospective single‐centre consecutive series; this design was appropriate to evaluate feasibility and early outcomes of a measurable balancing workflow in a real‐world setting while maintaining a high degree of technical standardization. Second, we did not include a contemporaneous control group; accordingly, the study was not designed to compare balancing philosophies or to establish superiority over alternative strategies. Third, the CPAK VI subgroup was smaller than the CPAK III subgroup, reflecting the relative frequency of constitutional valgus morphotypes in routine practice; therefore, subgroup comparisons should be considered exploratory rather than definitive. Fourth, intraoperative laxity quantification still relies on varus–valgus stress manoeuvres; although the robotic platform provides consistent millimetric readouts and the acquisition sequence was standardized, some variability in force application cannot be entirely excluded. Finally, the mean follow‐up of 26.5 months captures early stability and patient‐reported outcomes, but longer‐term studies are required to clarify durability and the relationship between objective laxity targets and late instability or revision.

## CONCLUSION

Intentional asymmetric soft‐tissue balancing in robotic‐assisted valgus TKA was associated with good early clinical outcomes and a reproducible reduction of lateral laxity while maintaining mild mediolateral asymmetry. These findings support the feasibility and reproducibility of a compartment‐specific laxity‐guided strategy in constitutional valgus knees, but do not establish superiority over alternative balancing philosophies or a causal relationship with clinical outcomes.

## AUTHOR CONTRIBUTIONS

Stefano G. Petrillo conceived the clinical idea of the study, performed the surgical procedures, provided the clinical case series, and contributed to data acquisition and surgical interpretation. Damiano Ardiri developed the study design and methodology, organized and curated the dataset, performed data analysis and interpretation, structured the scientific message of the study, and wrote the original draft of the manuscript. Matteo Marullo and Giampaolo Giordano contributed to data collection and critically revised the manuscript. Sergio Romagnoli provided senior supervision and critically revised the manuscript for important intellectual content. All authors reviewed and approved the final version of the manuscript.

## FUNDING INFORMATION

This work was supported and funded by the Italian Ministry of Health—‘Ricerca Corrente’. The article processing charge (APC) was funded by the Italian Ministry of Health—‘Ricerca Corrente’.

## CONFLICT OF INTEREST STATEMENT

Damiano Ardiri is Chief Executive Officer (CEO) and equity holder of the start‐up Caleb (medical device). Sergio Romagnoli and Stefano G. Petrillo are paid consultants for Zimmer Biomet. No patents are planned, pending, or issued that are directly related to this manuscript; no royalties or payments were received in relation to the present work. Zimmer Biomet had no role in the study design, data collection, analysis, interpretation of results or manuscript preparation. The other authors declare no conflicts of interest.

## ETHICS STATEMENT

Approved by the Comitato Etico Territoriale Lombardia 1 (CET Lombardia 1) (meeting date: 4 February 2026), promoted by IRCCS Ospedale Galeazzi–Sant'Ambrogio (approval/protocol code: ALLCCP). Written informed consent was waived by the Ethics Committee due to the retrospective design and use of de‐identified data.

## Supporting information


**Figure S1. Study flowchart (STROBE).** Flow diagram summarizing patient screening, exclusions, and final cohort allocation. Of 202 robotic‐assisted total knee arthroplasties screened during the study period, 144 cases were excluded and 58 constitutional valgus knees were included in the final analysis, comprising CPAK III and CPAK VI morphotypes.

**Supplementary File S1. STROBE checklist.** Completed STROBE checklist for observational studies, indicating where each reporting item is addressed in the manuscript.

## Data Availability

The data sets generated and/or analyzed during the current study are available from the corresponding author on reasonable request.
