# Supplementary figures and images for: Intentional asymmetric balancing in robotic‐assisted valgus TKA: Early outcomes and intraoperative laxity changes
Source: J Exp Orthop. 2026 Jun 22;13(2):e70810. doi: 10.1002/jeo2.70810 (PMC13285584; doi:10.1002/jeo2.70810)

# Study flowchart (STROBE)

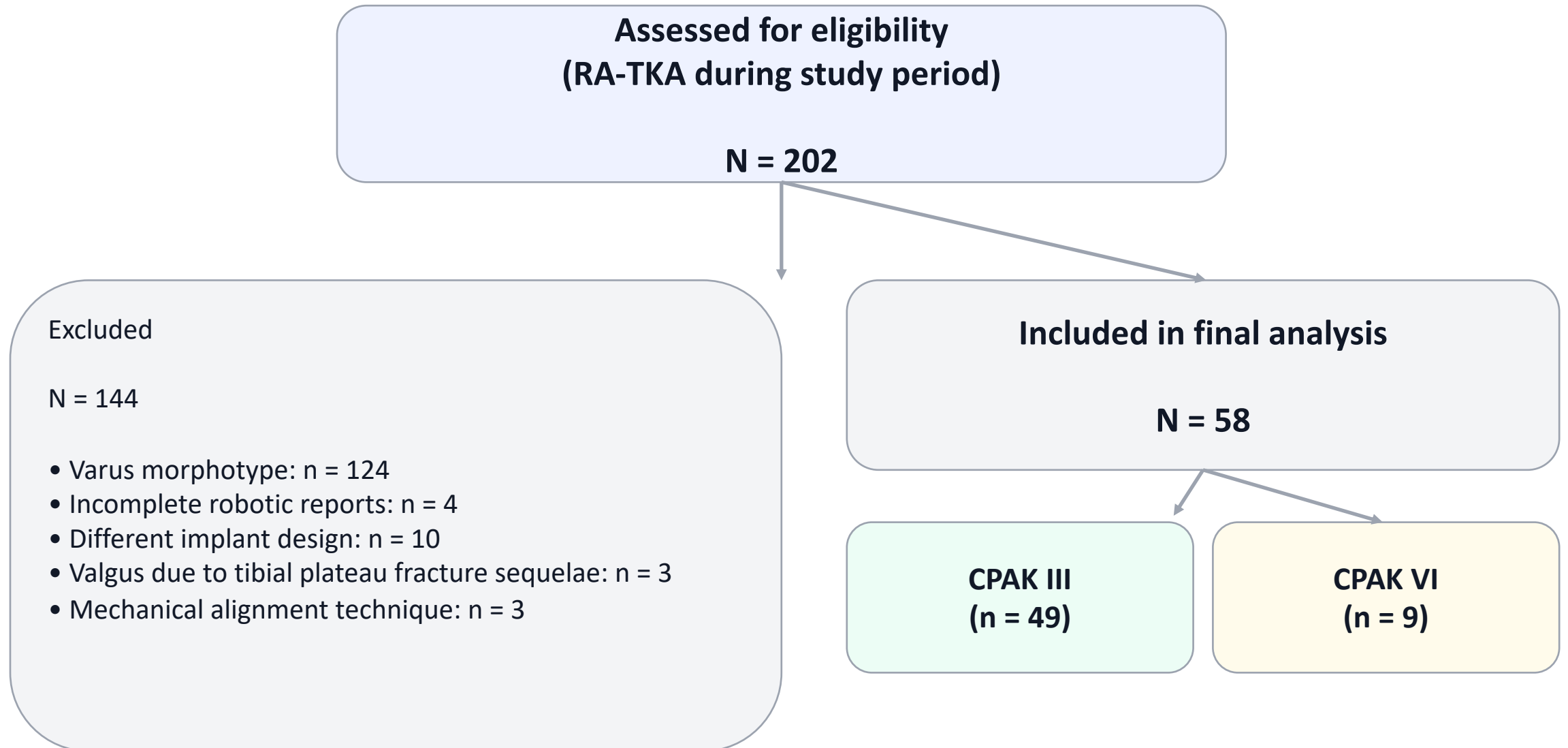

Supplement: Supplementary file 1 — Figure S1. Study flowchart (STROBE). Flow diagram summarizing patient screening, exclusions, and final cohort allocation. Of 202 robotic‐assisted total knee arthroplasties screened during the study period, 144 cases were excluded and 58 constitutional valgus knees were included in the final analysis, comprising CPAK III and CPAK VI morphotypes. [file JEO2-13-e70810-s002.pdf]
