# Supplementary material for: Intentional asymmetric balancing in robotic‐assisted valgus TKA: Early outcomes and intraoperative laxity changes
Source: J Exp Orthop. 2026 Jun 22;13(2):e70810. doi: 10.1002/jeo2.70810 (PMC13285584; doi:10.1002/jeo2.70810)
Supplement: Supplementary file 2 — Supplementary File S1. STROBE checklist. Completed STROBE checklist for observational studies, indicating where each reporting item is addressed in the manuscript. [file JEO2-13-e70810-s001.docx]

**Supplementary Table S1. STROBE checklist (retrospective cohort study)**

This study was conducted and reported in accordance with the STROBE statement for observational studies. The table below indicates where each STROBE item is addressed in the manuscript.

| Item | Recommendation | Where addressed in the manuscript |
| --- | --- | --- |
| 1a | Indicate the study’s design with a commonly used term in the title or the abstract. | Title and Abstract (structured). |
| 1b | Provide in the abstract an informative and balanced summary of what was done and what was found. | Abstract (Purpose/Methods/Results/Conclusion). |
| 2 | Explain the scientific background and rationale for the investigation being reported. | Introduction. |
| 3 | State specific objectives, including any prespecified hypotheses. | End of Introduction: Objectives and hypothesis. |
| 4 | Present key elements of study design early in the paper. | Materials and Methods: Study design, setting, and dates. |
| 5 | Describe the setting, locations, and relevant dates, including periods of recruitment, exposure, follow-up, and data collection. | Materials and Methods: Study design, setting, and dates. |
| 6a | Cohort: Give the eligibility criteria, and the sources and methods of selection of participants. | Materials and Methods: Participants. |
| 6b | Cohort: For matched studies, give matching criteria and number of exposed and unexposed. | Not applicable (no matching). |
| 7 | Clearly define all outcomes, exposures, predictors, potential confounders, and effect modifiers. Give diagnostic criteria, if applicable. | Materials and Methods: Surgical protocol; Outcomes and measurements. |
| 8 | For each variable of interest, give sources of data and details of methods of assessment (measurement). | Materials and Methods: Outcomes and measurements. |
| 9 | Describe any efforts to address potential sources of bias. | Materials and Methods: Bias. |
| 10 | Explain how the study size was arrived at. | Materials and Methods: Study size. |
| 11 | Explain how quantitative variables were handled in the analyses. | Materials and Methods: Statistical analysis. |
| 12a | Describe all statistical methods, including those used to control for confounding. | Materials and Methods: Statistical analysis (paired tests; subgroup analysis). |
| 12b | Describe any methods used to examine subgroups and interactions. | Materials and Methods: Statistical analysis; Results: Other analyses (CPAK III vs VI). |
| 12c | Explain how missing data were addressed. | Materials and Methods: Statistical analysis (complete-case; predefined exclusions). |
| 12d | Cohort: If applicable, explain how loss to follow-up was addressed. | Eligibility required minimum follow-up; flowchart reports exclusions. (Add explicit lost-to-follow-up count if applicable). |
| 12e | Describe any sensitivity analyses. | Not applicable (no sensitivity analyses reported). |
| 13a | Report numbers of individuals at each stage of study. | Results: Participant flow. |
| 13b | Give reasons for non-participation at each stage. | Results: Participant flow (reasons with counts). |
| 13c | Consider use of a flow diagram. | Figure S1 (this supplement). |
| 14a | Give characteristics of study participants (e.g., demographic, clinical, social) and information on exposures and potential confounders. | Results: Table 1 (Baseline characteristics). |
| 14b | Indicate number of participants with missing data for each variable of interest. | Results: STROBE 14b–c statement (baseline completeness). |
| 14c | Cohort: Summarise follow-up time (e.g., average and total amount). | Results: Table 1 (Follow-up mean ± SD). |
| 15 | Report numbers of outcome events or summary measures over time. | Results: Outcome data (Tables 2–4). |
| 16a | Give unadjusted estimates and, if applicable, confounder-adjusted estimates and their precision. | Results: Main results (deltas and P values; unadjusted). |
| 16b | Report category boundaries when continuous variables were categorized. | Not applicable (no categorization used). |
| 16c | If relevant, consider translating estimates of relative risk into absolute risk. | Not applicable. |
| 17 | Report other analyses done (e.g., subgroup analyses). | Results: Other analyses (CPAK morphotype subgroup). |
| 18 | Summarise key results with reference to study objectives. | Discussion: Key results. |
| 19 | Discuss limitations of the study, taking into account sources of potential bias or imprecision. | Discussion: Limitations. |
| 20 | Give a cautious overall interpretation of results considering objectives, limitations, multiplicity, and other evidence. | Discussion: Interpretation and comparison with literature. |
| 21 | Discuss the generalisability (external validity) of the study results. | Discussion: Generalisability. |
| 22 | Give the source of funding and the role of the funders for the present study and, if applicable, for the original study. | Title Page (Funding/COI/Data availability statements). |
